# Rapid neuroinflammatory changes in human acute intracerebral hemorrhage

**DOI:** 10.1002/acn3.50842

**Published:** 2019-07-13

**Authors:** Anan Shtaya, Leslie R. Bridges, Margaret M. Esiri, Joanne Lam‐Wong, James A. R. Nicoll, Delphine Boche, Atticus H. Hainsworth

**Affiliations:** ^1^ Neuroscience Research Centre, Molecular and Clinical Sciences Research Institute St. George’s, University of London London UK; ^2^ Department of Cellular Pathology St George's University Hospitals NHS Foundation Trust London UK; ^3^ Nuffield Department of Clinical Neurosciences Oxford University Oxford UK; ^4^ Clinical Neurosciences, Clinical & Experimental Sciences University of Southampton Southampton UK

## Abstract

**Objective:**

Spontaneous intracerebral hemorrhage (ICH) is the commonest form of hemorrhagic stroke and is associated with a poor prognosis. Neurosurgical removal of intracerebral hematoma has limited benefit and no pharmacotherapies are available. In acute ICH, primary tissue damage is followed by secondary pathology, where the cellular and neuroinflammatory changes are poorly understood.

**Methods:**

We studied histological changes in *postmortem* tissue from a cohort of spontaneous supra‐tentorial primary ICH cases (*n* = 27) with survival of 1–12 days, compared to a matched control group (*n* = 16) examined in corresponding regions. Hematoxylin–eosin and microglial (Iba1) immunolabelled sections were assessed at 0–2, 3–5, and 7–12 days post‐ICH.

**Results:**

Peri‐hematoma, the observed ICH‐related changes include edema, tissue neutrophils and macrophages from day 1. Ischemic neurons and swollen endothelial cells were common at day 1 and universal after day 5, as were intramural erythrocytes within small vessel walls. Activated microglia were evident at day 1 post‐ICH. There was a significant increase in Iba1 positive area fraction at 0–2 (threefold), 3–5 (fourfold), and 7–12 days post ICH (ninefold) relative to controls. Giant microglia were detected peri‐hematoma from day 5 and consistently 7–12 days post‐ICH.

**Interpretation:**

Our data indicate that neuroinflammatory processes commence from day 1 post‐ICH with changing microglial size and morphology following ICH and up to day 12. From day 5 some microglia exhibit a novel multiply nucleated morphology, which may be related to changing phagocytic function. Understanding the time course of neuroinflammatory changes, post‐ICH may reveal novel targets for therapy and brain restoration.

## Introduction

Spontaneous intracerebral hemorrhage (ICH) is a devastating cause of morbidity and mortality. The annual incidence ranges from 16 to 25 per 100,000 worldwide^1^, ^2^ with 40% mortality at 1 month.^2^ Risk factors include age (with significant increase in incidence above the age of 55^3^), male gender, hypertension (HTN), and African American or Hispanic race.^4^, ^5^, ^6^ The main age‐related vessel pathologies considered to underlie spontaneous ICH (primary ICH) are small vessel disease (arteriolosclerosis) and cerebral amyloid angiopathy (CAA).^7^, ^8^, ^9^

In addition, structural pathological processes associated with secondary ICH include ruptured aneurysm, vascular malformation, tumors, and hemorrhagic infarcts. Systemic factors (e.g., hematological malignancies, thrombocytopenia, and coagulopathies) are of etiologic importance and an associated ICH may be considered either primary or secondary brain hemorrhage.

Large spontaneous ICH causes instantaneous disruption of surrounding brain, termed mass effect, and is often rapidly fatal. ICH with mass effect and rise in intracranial pressure can be surgically evacuated to reduce the primary brain injury. Current treatment approaches to spontaneous ICH include (1) surgical evacuation of accessible hematoma, (2) controlling blood pressure, and (3) reversing anticoagulation, where applicable.^10^, ^11^, ^12^, ^13^ Although surgical evacuation has the potential to reduce brain tissue damage, by relieving local ischemia or removal of noxious chemicals,^14^, ^15^ class I evidence from clinical trials does not demonstrate improved outcomes.^10^, ^13^, ^16^, ^17^

The cellular mechanisms that underlie pathophysiological changes around the hematoma are poorly understood. Activation of inflammatory cascades and the release of cytotoxic mediators are implicated.^15^, ^18^, ^19^ These cause cell death and functional impairment, and are considered the hallmark of secondary brain damage.^20^ Understanding the inflammatory processes that follow ICH in humans may offer new therapeutic opportunities.

In healthy adult brain, microglia exhibit ramified morphology.^20^ In response to brain injury, microglia become activated and undergo morphological and functional transformations. Their cell bodies become enlarged, dense, with thicker processes, inflammatory proteins are upregulated, and the cells become migratory, proliferative, and phagocytic.^19^, ^20^, ^21^, ^22^, ^23^ Activated microglia can have either neurotoxic or neuroprotective properties and their overall effect depends on the type and severity of brain insult.^24^ Here, we hypothesized that microglial phenotype and morphology are altered in human spontaneous ICH.

We performed histopathological assessment of tissue obtained from subjects who died from spontaneous supra‐tentorial ICH. We examined tissue from the margin of the hematoma in 27 ICH cases, and analogous tissue samples from 16 control subjects. We report significant changes in the size and morphology of Iba1‐labelled microglia in human ICH.

## Materials and Methods

### Human tissue

This is a *postmortem* study of spontaneous ICH cases (*N* = 27, *M* = 13, *F* = 14, age range 19–90 years old, median = 59 years old; Table 1). Standard tissue blocks (1–2 blocks per case) were taken from tissue at the border of the hemorrhage and the average results for each case presented. A group of control subjects (*N* = 16) deceased due to non‐neuropathological cause (*M* = 11, *F* = 5, range 26–60, median 51 years old) were also examined in the same anatomical regions.

**Table 1:** Characteristics of the cases.

Number	Age/sex	ICH location (lobar vs. deep [basal ganglia/thalamus])	Time from hemorrhage to death (days)	Known relevant past medical history	Estimated ICH volume (mL)
1	86/M	Lobar	NA	AD	NA
2	90/F	Deep	NA	AD, HTN	NA
3	82/M	Lobar	NA	AD, IHD	NA
4	82/F	Deep	NA	AD	NA
5	90/M	Lobar	NA	None	NA
6	76/M	Lobar	NA	AD, CAA	NA
7	90/M	Lobar	NA	AD	NA
8	90/M	Lobar	NA	AD	NA
9	75/F	Deep	2	None	NA
10	40/F	Deep	2	Leukaemia	NA
11	52/M	Deep	4	None	NA
12	79/F	Lobar	12	None	42
13	56/F	Deep	12	None	NA
14	52/F	Deep	1	None	NA
15	58/F	Deep	1	None	27
16	59/M	Lobar	7	HTN	NA
17	78/M	Lobar	10	None	NA
18	30/M	Lobar	3	HTN	30
19	55/F	Deep	5	HTN	67.5
20	19/F	Lobar	8	pneumonia	188
21	53/F	Lobar	3	None	36
22	53/F	Lobar	1	None	67
23	27/F	Lobar	Within 1	Drug abuser, seizures	67
24	52/F	Deep	3	COPD, IHD	35
25	65/M	Deep	3	Alcoholism, Leukaemia	80
26	49/M	Lobar	3	Alcoholism, HTN	21
27	65/M	Deep	Within 1	HTN, DMII	316

ICH, intracerebral hemorrhage; AD, Alzheimer's disease; HTN, hypertension; CAA, cerebral amyloid angiopathy; COPD, chronic obstructive pulmonary disease; IHD, ischemic heart disease; DMII, diabetes mellitus type two; NA, not available.

**Figure 1:**
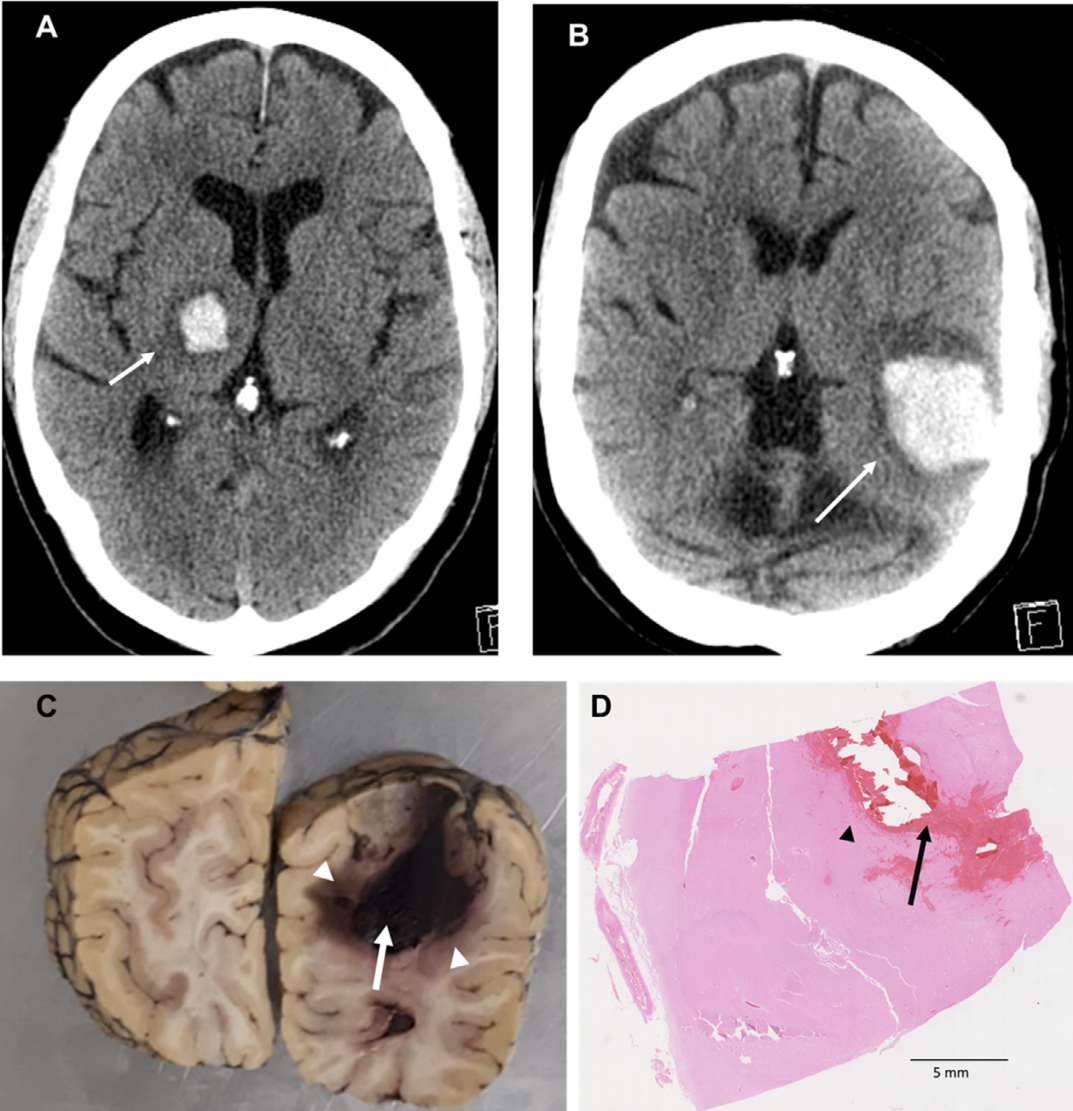
CT head and *postmortem* brain with ICH. (A) Axial CT head image shows right spontaneous deep thalamic hemorrhage. (B) Axial CT head image demonstrates spontaneous left temporo‐parietal (lobar) ICH. (C) Coronal section of a *postmortem* human brain with ICH. White arrow shows the hemorrhage and arrow heads show sampled areas. (D) H&E of an ICH human brain section. Arrow locates the hemorrhage. Arrow head shows the perilesional area that has been quantified and analysed. CT, computerized tomography; ICH, intracerebral hemorrhage; H&E, hematoxylin–eosin.

All ICH cases are of spontaneous supra‐tentorial hemorrhage (either lobar ICH or deep ICH within the basal ganglia or thalamus, Fig. 1A and B) with no known underlying structural abnormalities. *Postmortem* reports from the brain tissue banks were reviewed for location, dimension of the hematoma, intraventricular extension, brainstem hemorrhage, and comorbidities that include HTN, inflammatory diseases, Alzheimer’s disease pathology (Table 1). ICH volume was estimated from the *postmortem* report when available. The ABC/2 technique is a known and reliable bedside method for measuring ICH volume from the computerized tomography (CT) head which has been widely validated.^25^, ^26^ In the ABC/2 method, A is the greatest hemorrhage diameter by CT, B is the diameter perpendicular to A, C is the approximate number of CT slices with hemorrhage multiplied by the slice thickness.

### Ethics approval

Ethical approval was provided by BRAIN UK (Research Ethics Committee South Central Hampshire B, reference 14/SC/0098) for the cases sourced from St George’s University Hospital NHS Foundation Trust, North Bristol NHS Trust, and University Hospitals Plymouth NHS Trust. The cases provided by the Oxford Brain Bank and University of California, Irvine were covered by the ethics reference 12/EM/0028 from Health Authority Service, NRES Committee East Midlands‐Derby.

### Immunohistochemistry

Sections of formalin‐fixed paraffin embedded tissue were processed for hematoxylin–eosin (H&E) and immunohistochemistry as described previously.^27^, ^28^ Briefly, sections (6 µm) were de‐waxed and processed for standard immunohistochemical labelling. Endogenous peroxidase activity was blocked by exposure to H2O2 (3% v/v, aqueous solution) for 10 min. After high‐pressure heat‐induced antigen retrieval (30 sec, 125°C, in pH7.8 Tris‐citrate buffer), nonspecific binding was blocked with PBS supplemented with Triton‐X100 (0.1%) and BSA (3%) (PBT‐BSA) for 60 min at room temperature and sections were incubated with the primary microglial antibody Iba1 (1:800 in PBT‐BSA, MP‐290‐CR01, A.Menarini Diagnostics Ltd, Wokingham, UK) at 4°C overnight. Antibody labelling was visualized using a peroxidase‐conjugated secondary reagent (Envision^®^ kit, K500711, Agilent Technologies LDA, Oxford, UK) and diaminobenzidine chromagen, then counterstained for nuclear chromatin with Mayer’s hematoxylin. As a negative control, neighboring sections were treated with irrelevant primary antibody (rabbit anti‐sheep IgG; BD‐Pharmingen). Immunolabelling for the T‐cell marker CD3 was performed on a VENTANA BenchMark Immunohistochemistry automated staining machine in St George’s Hospital Cellular Pathology diagnostic service. The primary antibody is rabbit monoclonal IgG (clone 2GV6, Ventana‐Roche, Tucson, AZ) raised against a synthetic peptide from the C‐terminus of CD3 epsilon chain, found in T cells and NK cells.

For immunofluorescence, paraffin sections (6 μm thickness) were processed for labelling as in our previous work.^29^, ^30^ Heat‐induced antigen retrieval was performed using a Menarini‐Biocare decloaker, (120°C, 30 sec, in citrate buffer pH6). Nonspecific binding was blocked by incubation with 6% w/v BSA (Jackson Immunochemicals, Cambridgeshire, UK) in PBS‐T for 1 h at room temperature. Sections were incubated overnight in a humidified chamber at 4°C with the same primary antibody Iba1 (1:200) diluted in 6% w/v BSA in PBS‐T. Sections were incubated with appropriate secondary antibodies conjugated to Alexa546 or Alexa647, diluted 1:300 in 6% BSA in PBS‐T at room temperature for 1 h. After nuclear labelling with DAPI (20 min, 0.3 µmol/L in PBS‐T), sections were mounted and photographed with a Nikon A1R confocal microscope. Red fluorescence was viewed with 543 nm excitation and 545–575 nm emission bandwidth. Far red fluorescence was viewed with 640 nm excitation and 663–738 nm emission bandwidth. DAPI was viewed with 405 nm excitation and 425–475 nm emission bandwidth. Neighboring sections were processed identically in parallel, but with omission of primary antibodies.

### Histopathology assessment

**Figure 2:**
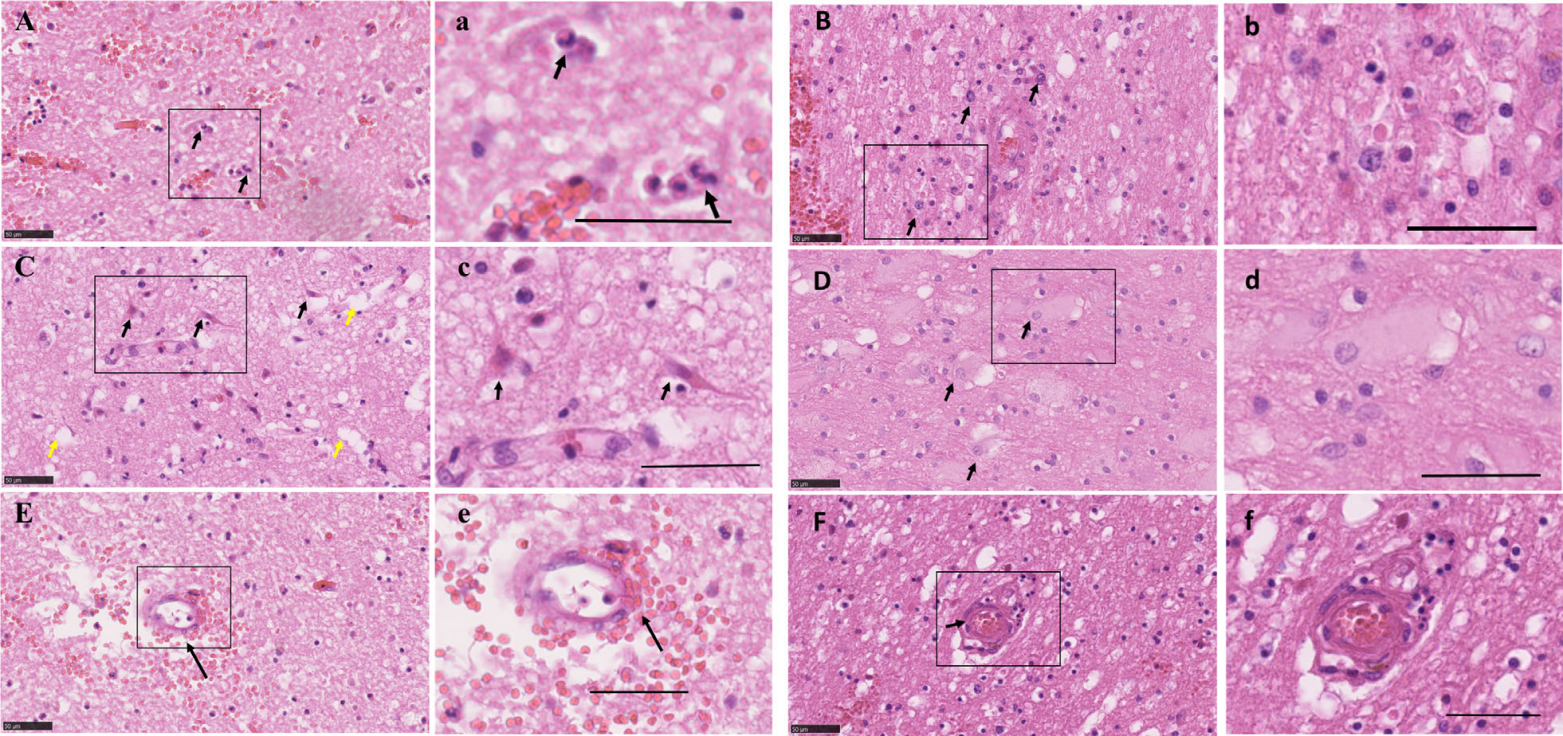
Acute cellular changes following spontaneous ICH in human. (A) Presence of neutrophils (arrows), (a) and at higher magnification. (B) Recruited macrophages, (b) and at higher magnification. (C) Red Neurons (black arrows) and vacuoles demonstrating edema (yellow arrows), (c) and at higher magnification. (D) Reactive astrocytes (white arrows), (d) and at higher magnification. (E) A small vessel with erythrocytes dissecting its wall (arrow), (e) and at higher magnification. (F) Thickened small vessel endothelial cells (arrow), (f) and at higher magnification. Scale bar 50 µm. ICH, intracerebral hemorrhage.

Microscopic examination was performed independently by two neuropathologists (L. R. B. and M. M. E.) on the H&E stained sections from all tissue blocks to identify the pattern of hematoma and peri‐hematoma changes. They were blind to clinical and autopsy data. Quantification, as absent or present, included the following features, neutrophils, macrophages, edema, red neurons, reactive astrocytes, small vessel intramural blood cells, and thickened small vessel endothelial cells (Fig. 2). Inter‐rater differences were reviewed and consensus achieved by discussion. Microscopic examination of T‐cells stained sections was performed blindly by neuropathologist (L. R. B.).

### Microglial quantification

Iba1‐immunolabelled slides were digitized at 20× magnification using a slide scanner (Hamamatsu WEB, Welwyn Garden City, Hertfordshire, UK). From the scanned slide, 10 images from the peri‐hematoma area were digitally acquired using NDP View software (Hamamatsu WEB) and analysed with Image J (Version 1.51j8, Wayne Rasband, NIH, Wisconsin). MaxEntropy macro filter was exclusively applied to threshold the images. Labelled‐area fraction (%AF) is reported as 100× (number of pixels positive for Iba1)/total number of pixels.

### Microglial morphology assessment

In ICH‐scanned sections, areas of maximal changes, defined as high cell density areas, were identified adjacent to the hematoma region. The morphology of Iba1‐labelled cells was assessed using the zoom in function of NDP.view2 Viewing software (Hamamatsu WEB) at 40× magnification. For comparison, similar areas in the same section were examined distant from the hematoma, and in sections from control cases.

**Figure 3:**
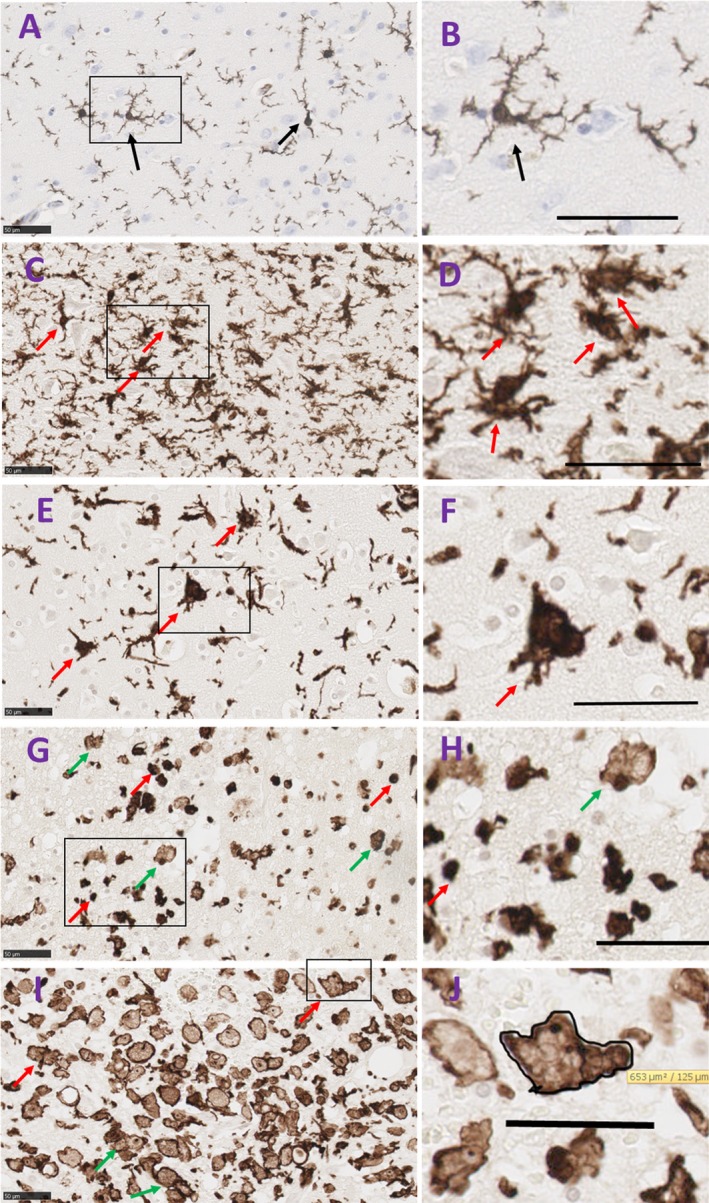
Morphologies of Iba1‐microglia in human brain tissue from control and spontaneous ICH cases. (A) Ramified microglia in a control subject defined by a small cell body which is <10 µm and multiple processes a few times longer than the cell body (arrow). (B) Higher magnification of a ramified cell, scale bar = 21 µm. (C) Activated microglia following brain hemorrhage, cell bodies are swollen, denser and larger than ramified microglia, processes are shorter and stouter, (arrows). (D) Higher magnification of activated microglia, scale bar = 50 µm. (E) Transitional microglia, processes fragments from dense cell body (arrows). (F) Higher magnification, scale bar = 50 µm. (G) Reactive microglia are typically small, spherical cells, but can also exhibit rod‐shaped and pleomorphic or amoeboid‐like morphologies; all lack ramified‐type processes (red arrows), amoeboid/phagocytic microglia are larger than reactive cells with fainter cytoplasm and dense nucleus located at the periphery of the cell body. They display various round and irregular shapes (green arrows). (H) Higher magnification of reactive microglia (red arrows) and amoeboid microglia (green arrows), scale bar = 50 µm. (I) Fusion stage of amoeboid microglia (green arrows) to form giant/large microglial cells where we noted bi/tri‐nucleate cells or fragmented nucleus or absence of nucleus, (red arrows). (J) Magnified picture of a bi‐nucleate giant microglia with an outlined boundaries to measure the cell surface area. Scale bar = 50 µm. ICH, intracerebral hemorrhage.

Briefly we describe physiological microglia that become activated, then progress to reactive passing through a transitional stage, with the reactive cells becoming amoeboid/phagocytic before some start fusing together in a stage we named “fusion stage” to form bi/tri‐nucleated giant cell (Fig. 3). Each microglial morphology was defined as follows:

*Ramified/ physiological microglia* possess a small (5–10 µm) oval cell body. The nucleus fills most of the soma leaving a very small volume of cytoplasm. Radiating from the soma are numerous processes of small diameter. These processes typically extend several times the diameter of the cell body in length, often branch, and usually exhibit a rough or spiny surface (Fig. 3A and B).^23^, ^31^, ^32^, ^33^

*Activated microglia* are swollen ramified cells and are characterized by a larger and denser cell body with shorter, and stouter processes (Fig. 3C and D).^31^, ^32^, ^33^

*Transitional microglia* are activated cells that appear to bead off their processes. They have dense cell body with an irregular shape (Fig. 3E and F). Some of these descriptions may be in part resembling dysmorphic microglia. However, dysmorphic microglia has been suggested to be due to microglial dysfunction as a result of aging^23^ which is not the case in our cohort.

*Reactive microglia* are typically small, spherical cells, but can also exhibit rod‐shaped and pleomorphic or amoeboid‐like morphologies. They lack ramified‐type processes (Fig. 3G and H).^31^, ^32^

*Amoeboid microglia* are spherical in shape, lack processes, and contain numerous phagocytic vacuoles (Fig. 3G and H). They are also defined as macrophage‐like morphology and are undistinguishable from recruited macrophages.

Early on, *reactive or amoeboid microglia adhere or fuse together to form* *giant nucleates cells* with no space between the cells. Some cells presented visible cell walls that were partly degraded in order to form giant cells. Nuclei are usually located in periphery of the cytoplasm (Fig. 3I). In this paper, for the first time, we are reporting the formation of a *giant/large cell* in the context of human ICH with cross‐sectional area ranges between 423–767 µm^2^.

The cross‐sectional area of microglial cell body was measured with the processes excluded in the case of physiological and activated microglia. This was achieved by drawing the margins of the cell body of Iba1‐labelled cells using the freehand region function of NDP.view2 Viewing software (Hamamatsu WEB) at 40× magnification (Fig. 3J). Ten cells per type per case were assessed.

### Statistical analysis

Descriptive analysis was performed to assess the normality of the data. For some of the analyses, the ICH cases were split according the time from hemorrhage to death including 0–2 days (*N* = 7); 3–5 days (*N* = 7) and 5–12 days (*N* = 5).

The data were parametric and the Student’s *t*‐test used to assess Iba1 AF (%) between ICH and control cohorts and within the ICH cases divided according age at death or the hemorrhage location. One‐Way ANOVA with Dunnett’s posthoc test was performed to analyse the temporal course of microglia and size of the microglial cell body between the different microglial morphologies identified. Chi‐squared test (χ^2^) analyzed whether HTN influenced the location of ICH. GraphPad Prism software was used to perform the statistical analysis, with *P*‐value considered significant when <0.05.

## Results

### Gross pathology of spontaneous ICH cases

Among the 27 ICH cases examined, deep ICH was recorded in 12 cases and lobar hemorrhage in 15 cases and (Table 1). Six were known hypertensives (three deep ICH and three lobar ICH; *P* = 0.76, χ^2^). Seven lobar ICH patients were diagnosed neuropathologically with Alzheimer’s disease. The hematoma dimensions were available for 12 cases (estimated volume 21–316 mL). Data about intraventricular extension of the ICH were available for 19 subjects of whom 14 (74%) had intraventricular extension of the ICH. Accurate data about time from ictus to death was available for 19 ICH cases (range 24 h–12 days). No AVM or other underlying structural abnormalities were known or identified on *postmortem* examination. The cause of death of all our cases was reported as spontaneous “acute ICH.”

### Histopathological changes in acute spontaneous ICH

On examination of H&E sections from ICH cases, neutrophils were present in the peri‐hematoma area as early as 1 day following the hemorrhage. Neutrophil infiltration was evident within 2 days of the ICH in most cases (63%), and in all cases by 5 days, up to 12 days, irrespective of whether the bleed was lobar or deep. Tissue macrophages, ischemic eosinophilic neurons (“red neurons”), swollen endothelia and reactive astrocytes were commonly observed at 1–2 days post‐ICH, and were universally present at 5–12 days (Fig. 2). Red blood cells within the wall of small vessels (intramural erythrocytes) were observed in 75% of subjects within days 0–2 and in all cases 5–12 days following the ICH (Fig. 2E and e). Edema was observed in the peri‐hematoma region from 1 day post‐ICH and subsequently in all ICH cases. Five out of eight ICH cases with Alzheimer’s disease had thickening of small vessel endothelial cells regardless of the time of death after ICH. All of the known hypertensive subjects had thickening of small vessel endothelial cells, regardless of time of death post‐ICH. None of these histopathological features was observed in the 16 control cases.

### Iba1 microglia following acute spontaneous ICH

**Figure 4:**
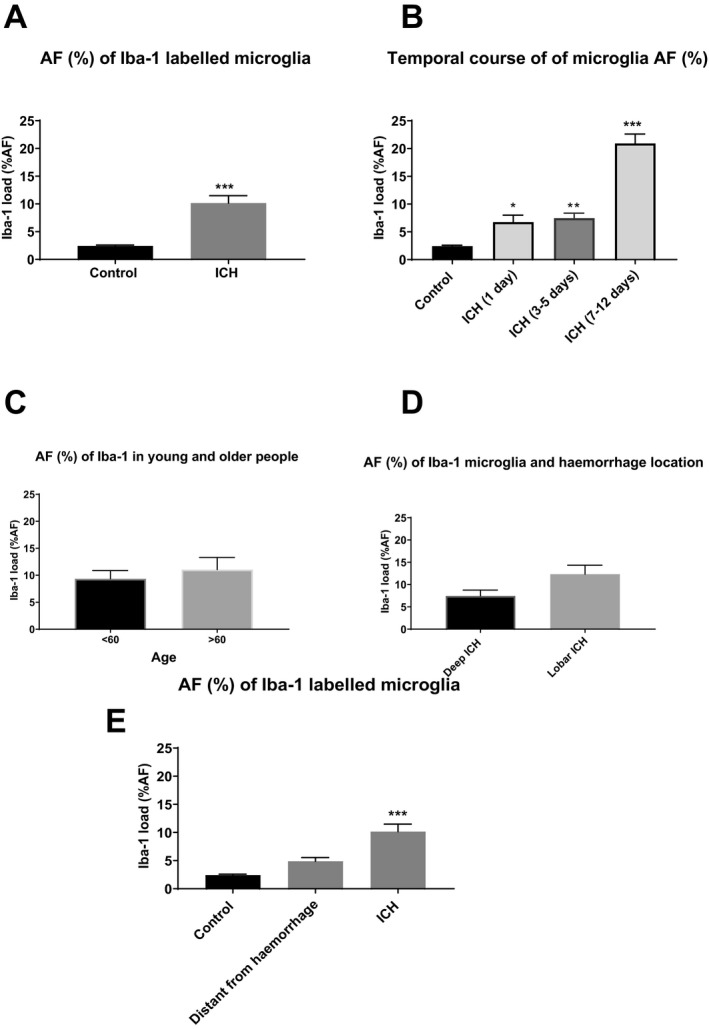
Iba1 microglia in control and ICH subjects. (A) Iba1 %AF (%) microglia increased significantly in 27 ICH subjects when compared to 18 controls (*P* < 0.0001). (B) Iba1 % increased significantly in subjects who died 1 day after ICH and 3–5 days after ICH. The greatest change was observed in subjects dying 5–12 days post‐ICH. (C) Iba1 microglial expression did not change when comparing those above 60 years old (*n* = 14) with those below 60 years old (*n* = 13; *P* = 0.54). (D) A trends toward increase was detected for Iba1 load in lobar ICH (*n* = 15, 12.3%) when compared to deep ICH (*n* = 12, 7.5%; *P* = 0.06). *P < 0.05, **P < 0.01, ***P < 0.00. (E) Iba1 microglia in control and ICH subjects (peri‐hemorrhage and distant from hemorrhage). Iba1 %AF (%) microglia increased significantly in 27 ICH subjects when compared to 18 controls (*P* < 0.0001). There was no significant difference in Iba1 load when comparing distant from hemorrhage (a lobe from hemorrhage or equivalent contralateral anatomical area) quantification to control (*n* = 13, 4.9% vs. 2.4% in controls, *P* = 0.52). There was a significant increase in Iba1 %AF in ICH when compared to distant from hemorrhage (10.16% vs. 4.9% *P* = 0.007). One‐way ANOVA with Bonferroni’s posthoc test. *P < 0.05, **P < 0.01, ***P < 0.001. ICH, intracerebral hemorrhage.

**Figure 5:**
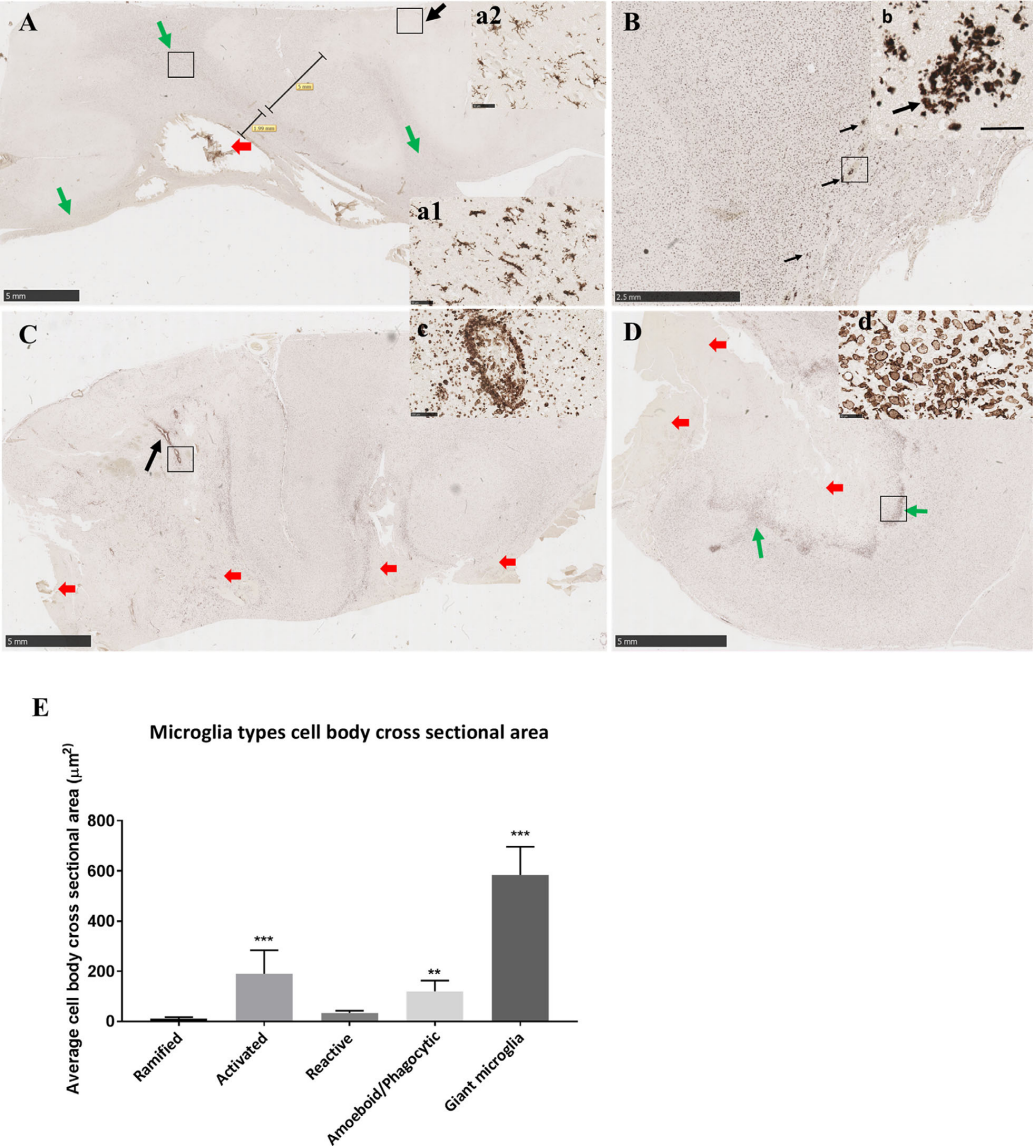
Representative images of morphological microglial changes over time. (A) Day 1 post‐ICH. Reactive and amoeboid/phagocytic microglia were observed within 2 mm of the hemorrhage (red arrow shows the hemorrhage site). Activated microglia were seen within 5 mm beyond the initial 2 mm zone around the hemorrhage and in white matter tracts (green arrows), (a1) shows a higher magnification of activated microglia. Ramified microglia were observed far from the bleed (black arrow) and (a2) shows a higher magnification of ramified microglia distant from the bleed. Panel (A) Scale bar = 5mm. Panel a1 scale bar = 50 µm. Panel a2 scale bar = 50 µm. (B) Day 3 post‐ICH. Mainly reactive and amoeboid/phagocytic microglia cells and occasional fusion microglia were observed within 2–3 mm of the hemorrhage site. We observed clusters of reactive and amoeboid microglia (black arrows) within 2–3 mm of the hemorrhage. Scale bar = 2.5mm. (b) Magnified picture of microglia clusters described in B. Scale bar = 50 µm. (C) Day 5 post‐ICH. Activated, transitional, reactive, amoeboid, and a few giant microglia were observed within 2–3 mm of hemorrhage. We noted clusters of reactive/amoeboid microglia around medium‐sized blood vessels (black arrow) and at higher magnification in panel (c). Reactive and transitional microglia were seen in the other parts of tissue section. No ramified microglia was observed. Red arrows represent hemorrhage areas. Panel (C) Scale bar = 5mm. Panel (c) scale bar = 50 µm. (D) Day 7 post‐ICH. Reactive, amoeboid/phagocytic, microglia that started fusing and giant microglia/macrophages were observed within 2–3 mm of the hemorrhage site (green arrow) and higher magnification presented in panel (d). Reactive and transitional microglia were seen around the hemorrhage site. No ramified microglia was observed. Red arrows represent hemorrhage areas. Panel (D) Scale bar = 5mm. Panel (d) scale bar = 50 µm. (E) Quantification of the cell body cross‐sectional area in the different microglial morphologies, **P < 0.01; ***P < 0.001. ICH, intracerebral hemorrhage.

Neighboring sections labelled with a microglial marker (Iba1) showed microglial cells adjacent to the hematoma (Figs. 4, 5). Semi‐quantitative assessment of the Iba1‐positive area fraction (%AF) confirmed fourfold greater extent of microglial labelling in ICH compared to controls (10.2 ± 1.3 %, 2.4 ± 0.2%, respectively, *P* < 0.0001, Fig. 4A). There was significant change in AF% with time post‐ICH (*P* < 0.05, Fig. 4B). At days 3–5, Iba1 AF (%) increased significantly in comparison with controls (*P* < 0.01) and was nine times higher in subjects dying between 5 and 12 days post‐ICH (*P* < 0.001; ICH: 20.92 ± 0.1.7% vs. controls: 2.4 ± 0.2%).

To test for age dependence, we dichotomized the cohort to those aged <60 years and those aged 60 or more. There was no significant difference in Iba1 AF (%) between these age groups (Fig. 4C). We observed a trend toward greater Iba1 AF (%) in lobar ICH (12.3 ± 2.0%) compared with deep ICH (7.5 ± 1.3%, *P* = 0.06; Fig. 4D).

### Temporal course of morphological changes of Iba1‐microglia

In Iba1‐labelled sections transitional, reactive, and amoeboid microglia were observed within 2 mm of the hematoma from 24 h post‐ICH (Fig. 5). Activated microglia were observed in the neighboring 5 mm and white matter tracts (examples in Fig. 5a1). Ramified microglia were also seen in the same cases, adjacent to the hematoma and in locations distant from the hematoma (Fig. 5a1, a2).

**Figure 6:**
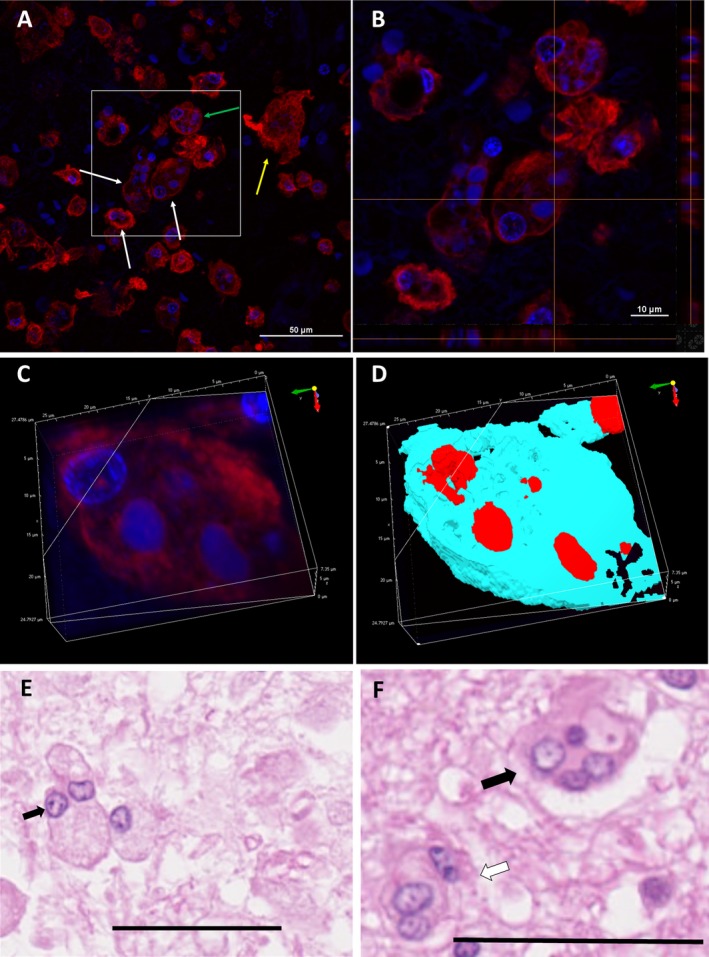
Confocal and H&E micrographs of Iba1‐labelled microglia demonstrating the giant/large morphology. (A) Merged micrograph of far red Iba1‐labelled microglial cells (red) and DAPI‐stained cell nuclei (blue). White arrows show multinucleate (often bi or tri)‐nucleated giant/large microglia with: green arrow highlighting a giant cell with a nuclei and fragments of nuclei by the border of the cell membrane. Yellow arrow shows a giant cell with no nuclei. Scale bar = 50 µm. (B) A magnified confocal orthogonal micrograph from micrograph (C). Scale bar = 10 µm. (C and D) 3D confocal micrographs demonstrating giant multinucleate microglia. (E) H&E micrograph shows a bi‐nucleate microglia (arrow), there is also another neighboring cell that could be in the process of fusion. Scale bar = 50 µm. (F) H&E micrograph demonstrate multinucleate giant microglia (black arrow) and bi/tri‐nucleate microglia (white arrow). Scale bar = 50 µm. H&E, hematoxylin–eosin.

At 3–5 days post‐ICH, reactive and amoeboid microglial cells remained abundant 2–3 mm of the hemorrhage, as well as clusters of Iba1 cells (Fig. 5B and b). In addition, some large bi‐ or tri‐nucleated Iba1‐positive cells were seen. These were much larger than the other microglial cells, with an overall amoeboid morphology, that we have termed giant microglia (examples in Fig. 6). Ramified microglia were less evident adjacent to the hematoma, while activated and transitional microglia were seen in the rest of the tissue section.

From day 5 post‐ICH, reactive, amoeboid microglia were common within 2–3 mm of the hemorrhage (Fig. 5C and D). Clusters of amoeboid microglia were observed around medium‐sized blood vessels (Fig. 5C and c). Bi‐/tri‐nucleate and occasional multinucleate giant microglia were also commonly seen adjacent to the hematoma. Within the rest of the tissue section, reactive and transitional microglia were observed, while ramified microglia were sparse or absent.

The presence and morphology of bi‐ and tri‐nucleated giant microglia were confirmed by orthogonal and 3D confocal micrographs of Iba1‐labelled sections using immunofluorescence (Fig. 6).

### CD3‐labelled T cells following ICH

CD3‐immunopositive T cells were seen in all cases (control and hemorrhage) in blood inside vessels. They were also seen in the hemorrhage cases within the blood clots.

T cells were also seen in all cases (control and hemorrhage) around blood vessels in the Virchow‐Robin space (Fig. S1). The significance of this is uncertain but the numbers of T cells were few and not noticeably different between control and hemorrhage cases. It could be that they represent a response to old nonspecific minor insult (they were often associated with a few hemosiderin‐laden macrophages).

None of the controls showed parenchymal T cells. Assessment of the brain parenchyma in the hemorrhage cases was done carefully to exclude T cells which were just part of the blood clot. In 11 out of 12 acute hemorrhage (1–3 days) cases, there were no parenchymal T cells; the remaining case (at 3 days) showed appreciable T cells (Fig. S2). In later hemorrhage cases, scanty parenchymal T cells were present in one case each at 7 and 8 days. One case at 12 days (without obvious organisation – vascularisation – of the clot) showed only a few parenchymal T cells. Another case at 12 days (with clear organisation of the clot) showed moderate T cells in the organising tissue and the brain parenchyma (Fig. S3).

### The cross‐sectional area of microglia cell bodies

We measured the cross‐sectional area of microglia cell bodies from the different morphologies identified. The surface area of activated microglia was significantly larger than ramified microglia (189.9 ± 28.3 µm^2^, 11.23 ± 1.8 µm^2^ respectively *P* < 0.001, Fig. 5E). The cross‐sectional area of reactive microglial cells was not significantly different from ramified microglia (Fig. 5E). Amoeboid cells had significantly larger cell bodies than ramified cells (*P* = 0.004). However, the largest among all were the giant/large microglial cells with a mean of 584 ± 33.8 µm^2^ (minimum 423 µm^2^ and maximum 767 µm^2^ *P* = 0.0001). These measurements were repeated with 30 day interval and showed good intra‐rater agreement (κ = 0.88).

## Discussion

We studied the histological changes in spontaneous ICH and their temporal course in 27 spontaneous ICH cases. Some of the included subjects who presented with ICH may have had undiagnosed HTN, as the proportion of known hypertensive subjects in our study is lower than those reported in the literature.^34^, ^35^ Seven ICH cases had neuropathological diagnosis of AD and in these individuals CAA may be the cause of ICH.^36^ Intraventricular extension of ICH, a factor associated with poor prognosis,^37^ was reported in 74% of our cases, consistent with the time from hemorrhage to death of our cohort.

Edema in the peri‐hematoma region was detected from day 1 post‐ICH and was a consistent feature in all ICH cases. Our observation is consistent with peri‐hematoma edema reported from head scans of ICH patients within 1 day of the hemorrhage and in association with poorer outcome.^19^, ^38^ Red neurons indicative of ischemic neuronal damage were present within the peri‐hematoma area in most (88%) of the subjects within 3 days of the ICH and up to 12 days post‐ICH. Neutrophils are widely considered to be the earliest leukocyte subtype to infiltrate into the hemorrhagic brain. As neutrophils are known to produce reactive oxygen species which cause neurotoxicity and contribute to brain damage in animals,^39^ their early presence and leakage of their contents is associated with brain tissue damage.^20^ The temporal course of neutrophil infiltration in human ICH is relatively unexplored.^19^ In our human cohort, we observed neutrophil infiltration within 2 days of the ICH in most cases and in all cases by 5 days post‐ictus, with tissue neutrophils evident up to 12 days. Notably, neutrophils were less numerous than macrophages throughout the course of the post‐ICH inflammatory response in our cohort. Tissue macrophages were observed in most of the cases (88%) from 2 days post‐ICH, and then in all subjects. We saw erythrocytes within the wall of small vessels with arterial appearance, as well as abnormal and swollen endothelial cells of small vessels in most of our ICH cases from day 2. This suggests active involvement of small vessels in the reaction following ICH and perhaps a role in trafficking or clearance of hematoma‐derived debris.

Lymphocytes, in particular T cells, are known to enter brain tissue and have been reported to accumulate after ischemic stroke.^40^ Subtypes of T cells (including NK cells) that invade brain tissue are likely to contribute to local inflammation and tissue damage. In sections labelled for the pan‐selective T‐cell marker CD3, T cells were routinely seen in blood inside vessels which is expected as T cells are a normal blood complement. We also observed some T cells in Virchow‐Robin spaces. The significance of this is uncertain but the numbers of T cells were few and not noticeably different between control and hemorrhage cases. It could be that they represent a response to old nonspecific minor insult (they were often associated with a few hemosiderin‐laden macrophages). None of the controls showed parenchymal T cells. Out of 12 ICH cases (1–3 days post‐ICH) assessed only one case showed some T cells. We also noted scanty T cells in two cases (days 7 and 8 post‐ICH) and one case (12 days post‐ICH). Our data suggest that the main response we see after ICH is mainly mediated by microglia. Furthermore, future studies may explore any involvement of T cells following brain hemorrhage.

Secondary brain injury following ICH involves activation of inflammatory pathways.^19^, ^21^ Microglia are the resident immune cells of the brain and are the first glial cells to react to brain hemorrhage.^21^, ^22^, ^41^ There is very limited information concerning the microglial response in terms of magnitude, cell morphology and temporal course in spontaneous human ICH. In human ICH brain tissue labelled with the microglial marker Iba1^42^, ^43^, ^44^, ^45^ and we observed increased Iba1 expression in the peri‐ICH region. Iba1 expression was increased as early as 1 day post‐ICH, in contrast to animal studies where activated microglia become apparent at day 3 after experimental ICH.^19^, ^46^

We did not observe a pronounced influence of patient age, when we compared Iba1‐positive AF% in people under 60 versus those over 60 years of age. We noted a trend for extensive Iba1‐positive AF% in lobar ICH than in deep ICH cases. If this is confirmed it may reflect differences in the brain location of the hemorrhage or in the underlying vascular pathology.

Microglial activation is defined as the process by which microglia cells change shape, molecular signature, and cellular physiology.^47^ In brain tissue of human ICH, we have observed various microglial morphological changes including activated, reactive, amoeboid microglia, as previously reported following injuries.^23^, ^31^, ^32^, ^45^, ^48^, ^49^, ^50^ The morphology of microglia after hemorrhage was defined more than 20 years ago.^31^ We have modified this previous classification to include morphologies observed in our ICH cohort. Microglia with ramified morphology, assumed to be in a physiological state, are extremely motile cells^51^ and key regulators of neuronal and synapse function, actively participating to the synaptic activity in the adult brain.^52^ Morphological changes are likely to reflect the changes in microglial function. Therefore, based on the timescale and the morphological changes observed (number and size of processes, cell body surface), we hypothesize the following progression of microglial fate. First, ramified microglia become activated following ICH, likely due to the blood–brain barrier breakdown progressing through a transitional stage before they become reactive, then amoeboid, reflecting increased phagocytic activity to clear the brain from highly toxic components such as blood cells and dead cells.

We report bi‐ or tri‐nucleated giant amoeboid microglia in the peri‐hematoma zone, from 5 days post‐ICH, which we believe is a novel finding (see Fig. 6). Multinucleated giant cell microglia have been previously reported in association with viral encephalitis and HIV,^53^, ^54^, ^55^, ^56^, ^57^ but not to our knowledge in human ICH. We speculate that these giant cells represent fusion of neighboring activated microglia to attain a greater phagocytic capacity (as previously reported for multinucleated giant cells^57^). Alternatively, they may reflect dysfunctional, dying microglia that are overwhelmed by the inflammation and cytotoxic environment adjacent to the hematoma. A third possibility is that rapid proliferation of microglia in response to acute ICH could generate abnormal, multinucleated cells.

A limitation of our study is that the Iba1 marker, though commonly used to identify microglia in histological studies, does not discriminate subtypes of microglia or distinguish microglia from monocyte/macrophages. This becomes a pertinent issue in a setting like ICH with robust infiltration of peripheral leukocytes and we are exploring other markers, to distinguish distinct subtypes of microglia and macrophages, for a future report.

We conclude that neuroinflammatory changes occur early after ICH in human patients, with tissue invasion by leukocytes (neutrophils, macrophages) from day 1. Microglia were significantly activated from day 1 post‐ICH, with a characteristic temporal course and changes in cell morphology up to 12 days post‐ictus. Bi‐ or tri‐nucleated giant microglia, possibly resulting from microglial fusion, are a noticeable feature around the hematoma from day 5 and further study is required to understand the functional role of these cells in ICH.

## Conflict of Interest

The authors declare that they have no conflict of interest.

## Supporting information


**Figure S1.** Control case. T cells around a vessel in the Virchow‐Robin space. Immunohistochemistry for CD3, 20× magnification, scale bar = 100 µm.

**Figure S2.** Hemorrhage case at 3 days. Brain parenchymal T cells. Immunohistochemistry for CD3, 80× magnification, scale bar = 20 µm.

**Figure S3.** Hemorrhage case at 12 days. T cells in the brain parenchyma and associated with vascularisation (organisation/repair) response. Immunohistochemistry for CD3, 20× magnification, scale bar = 100 µm.
